# Prion protein oligomers cause neuronal cytoskeletal damage in rapidly progressive Alzheimer’s disease

**DOI:** 10.1186/s13024-021-00422-x

**Published:** 2021-02-22

**Authors:** Mohsin Shafiq, Saima Zafar, Neelam Younas, Aneeqa Noor, Berta Puig, Hermann Clemens Altmeppen, Matthias Schmitz, Jakob Matschke, Isidre Ferrer, Markus Glatzel, Inga Zerr

**Affiliations:** 1 Department of Neurology, University Medicine Goettingen and German Center for Neurodegenerative Diseases (DZNE), 37075 Goettingen, Germany; 2 Institute of Neuropathology, University Medical Center Hamburg-Eppendorf (UKE), 20246 Hamburg, Germany; 3 Biomedical Engineering and Sciences Department, School of Mechanical and Manufacturing Engineering (SMME), National University of Sciences and Technology (NUST), Islamabad, Pakistan; 4 Department of Neurology, Experimental Research in Stroke and Inflammation (ERSI), University Medical Center Hamburg-Eppendorf, 20246 Hamburg, Germany; 5 Institut de Neuropatologica, Servei Anatomia Patològica, IDIBELL-Hospital Universitari de Bellvitge, Universitat de Barcelona, Carrer Feixa LLarga sn, 08907, Hospitalet de LLobregat, CIBERNED, Barcelona, Spain

**Keywords:** Rapidly progressive Alzheimer’s disease, rpAD, Growth arrest specific proteins, GAS, Growth arrest specific 2 like 2, G2L2, Prion protein oligomers, PrP^C^, Co-immunoprecipitation, Cytoskeleton, Actin, Tubulin

## Abstract

**Background:**

High-density oligomers of the prion protein (HDPs) have previously been identified in brain tissues of patients with rapidly progressive Alzheimer’s disease (rpAD). The current investigation aims at identifying interacting partners of HDPs in the rpAD brains to unravel the pathological involvement of HDPs in the rapid progression.

**Methods:**

HDPs from the frontal cortex tissues of rpAD brains were isolated using sucrose density gradient centrifugation. Proteins interacting with HDPs were identified by co-immunoprecipitation coupled with mass spectrometry. Further verifications were carried out using proteomic tools, immunoblotting, and confocal laser scanning microscopy.

**Results:**

We identified rpAD-specific HDP-interactors, including the growth arrest specific 2-like 2 protein (G2L2). Intriguingly, rpAD-specific disturbances were found in the localization of G2L2 and its associated proteins i.e., the end binding protein 1, α-tubulin, and β-actin.

**Discussion:**

The results show the involvement of HDPs in the destabilization of the neuronal actin/tubulin infrastructure. We consider this disturbance to be a contributing factor for the rapid progression in rpAD.

**Supplementary Information:**

The online version contains supplementary material available at 10.1186/s13024-021-00422-x.

## Background

Alzheimer’s disease (AD) is the most prevalent form of dementia, affecting over 30 million people worldwide and representing 60–70% of all dementia cases [1]. Sporadic AD (spAD) and familial AD (FAD) cases are classically characterized by a progressive cognitive decline with an average post-diagnosis survival of eight years [2]. However, some AD cases mimicking rapidly progressive dementias, i.e. with accelerated progression rates and steep cognitive decline, have been reported over the past years [3–5]. These rapidly progressive Alzheimer’s disease (rpAD) cases are reported to have a shorter post-diagnostic survival time as well (shorter than four years) compared to those of typical AD cases (eight years on average) [6–9]. Due to their rapid progression, these cases are often initially misdiagnosed as prion diseases [10]. The physiological alterations responsible for the accelerated disease course in rpAD patients are poorly understood. No genetic linkage has been found between rpAD and any of the well-established autosomal pathogenic mutations in the *PSEN1*, *PSEN2, APP*, or *PRNP* genes coding for presenilin isoforms, the amyloid precursor protein, and the cellular prion protein, respectively [11, 12]. Patients with rpAD exhibit a distinct profile of CSF biomarker for neurodegeneration. A study from Abu Rumeileh et al. (2017) reported a remarkably higher CSF tau level in rpAD cases (median = 1223 pg/mL, *n* = 44) compared to that of spAD (median = 697 pg/mL, *n* = 45) [13, 14]. However, CSF levels of phosphorylated tau (p-tau), Aβ_40_, Aβ_42_, α-synuclein and YKL-40 do not show significant differences between rpAD and spAD patients [15, 16]. Additionally, in a large scale study comprising over 300 patient samples, the CSF p-tau/tau ratio in rpAD patients was found to be lower than that in spAD [17]. Other features differentiating rpAD from spAD include younger age of onset, lower frequency of the *APOE4* allele [12], increased serum levels of proinflammatory cytokines in rpAD (G-CSF, TNFα, IL-6 and IL-13) [18], and a higher incidence of rpAD cases testing positive for the 14–3-3 protein in the CSF [12]. Although not directly comparable to our study due to difference in definition of rapid progression (survival in our study, cognitive decline in study of Ba et al., 2017); rpAD patients  are also reported to exhibit region-specific hypometabolism in [18F]fluorodeoxyglucose-positron emission tomography by  Ba et al., (2017) [17]. Moreover, a higher abundance of low molecular weight (LMW) amyloid oligomers has been associated uniquely with AD cases showing accelerated progression rates, thus suggesting their possible involvement in the rapid progression [19]. In a nuclear magnetic resonance study, Qiang and coworkers have reported unique Aβ_40_ fibrils prepared by seeded growth from extracts of brain cortex tissues of rpAD patients. However, no physiological relevance of theses variants has yet been studied further [20]. In an earlier study, we described the presence of high molecular weight oligomers of the cellular prion protein (PrP^C^) in the frontal cortex, specifically in patients with a rapidly progressive form of AD [8]. Physiological involvement of PrP^C^ in the progression of AD pathology is well described [21]. Previous studies have emphasized the role of PrP^C^ at the neuronal surface in neurotoxic signaling utilizing cAMP/PKA or Erk activated Fyn kinase pathway [22, 23], whereas more neuroprotective functions seem to be mediated by extracellular fragments of PrP^C^, which sequester amyloid oligomers and may inhibit their spread and toxicity [24, 25]. However, the physiological relevance of the prion protein oligomers consistently identified in the brains of rpAD patients has not been explored.

In the course of AD, the brain tissue undergoes many systemic changes including, more prominently, the development of neurofibrillary tau tangles [2], amyloid-β senile plaques [26, 27], synaptic damage [28–30] and dysregulation of the cytoskeletal machinery [31]. The cytoskeleton plays a crucial role in the growth and function of the neurons [32]. Effective transport systems in the neuronal cytoplasmic processes, the intracellular organization of organelles, and a degree of signal transduction are primarily orchestrated by the microtubule system in the neurons [33–37]. This microtubular system works with the association of dyneins [38], kinesins [39], spectrins, plakins and spectraplakins [40]. Neurons also rely on a more transient actin system for establishing the dendritic spines, extending the neurites, and for inter-neuronal connections, in cooperation with the associated proteins such as integrins, cofilin and formin [41–44]. Various cytoskeletal anomalies are associated with Alzheimer’s disease as well. Synaptotoxicity in AD is attributed to the malfunctioning in the Rho-associated protein kinase (ROCK) and cofilin-actin machinery [45, 46], along with altered drebrin / Ca^2+^ /F-actin-controlled microtubule dynamics [47]. Amyloid-β has also been reported to alter the dendritic morphology in hippocampal neurons [48]. Microtubule / kinesin-mediated axonal vesicle transport is also affected in the course of the disease, caused by detachment of tau from microtubule filaments after hyperphosphorylation [38, 49]. Drummond et al. (2017) described cytoskeletal proteins differentially associated with the amyloid plaques in rpAD and spAD. The expression of the POTE ankyrin domain family member E, tubulin polymerization-promoting protein, and tubulin alpha-4A chain was found significantly increased in the cortical amyloid plaques of rpAD patients, encouraging further studies of cytoskeletal proteins in rpAD brains [50]. PrP^C^  is  also  reported to interact with cytoskeletal components including cofilin [51], actin [52], and tubulin resulting in the inhibition of microtubules assembly [53–55]. In the current study we aimed to identify cytoskeletal proteins interacting with the previously described high density PrP^C^ oligomers (HDPs) in the brains of rpAD patients, thereby gaining insight into the pathophysiological relevance of HDPs in rpAD.

## Methods

### Sample collection and processing

Patient material was obtained after the approval of local ethics committees at the University Medical Center, Goettingen. Frontal cortex samples from patients with spAD (*n* = 10), rpAD (*n* = 9), dementia with Lewy bodies (DLB) (*n* = 3), age-matched non-demented controls (Con) (n = 10) and other rapid dementias including small vessel disease (SVD) (n = 3), rapidly progressive dementia with Lewy bodies (rDLB) (*n* = 2), and dementia with frontotemporal lobar degeneration (DFTL) (n = 3) cases were provided by the brain bank of the Institute of Neuropathology (HUB-ICO-IDIBELL Biobank) and the biobank of the Hospital Clinic-IDIBAPS, Spain, according to their biomedical study legislation (Ley de la Investigación Biomédica 2013 and Real DecretoBiobancos, 2014). Frontal cortex samples from patients with sporadic Creutzfeldt-Jakob disease (sCJD) subtypes (MM1: *n* = 6, MM/MV2: n = 6, VV2: n = 6) were obtained from the Department of Neurology at the University Medical Center, Göttingen, Germany. The rpAD patients met the current selection criteria for rpAD [6–9, 11, 12, 19]. These inclusion criteria are as follows:

1. Initial classification as prion diseases based on clinical features
2. Presence of typical AD pathological features, i.e., higher Braak stages
3. Post diagnostic survival time (disease duration) shorter than four years
4. Exclusion of other forms of rapid progressive dementias and copathologies e.g. prion diseases, extensive Lewy body pathology, vascular damage or tumors based on postmortem neuropathological examination
5. Absence of a family history suggestive of familial AD

Cortex samples of the non-demented controls exhibited only mild AD pathology (Braak stage I – II). Both, rpAD and spAD samples presented AD pathologies ranging from Braak stage IV to VI. Likewise, the sCJD subtypes cohort (used as positive controls for the PrP-oligomers) presented classical profiles for prionopathies. The rDLB samples had co-pathology of AD, argyrophilic grain disease (AGD), tauopathy, or progressive supranuclear palsy (PSP). Patients from the DFTL cohort also showed features of motor neuron disease (MND) and TDP-43 pathology. All of the vascular pathology patients (SVD) co-exhibited higher stages of AD pathology. Further details including neuropathological assessment and the processing of the samples for different analyses in the current study are reported as supplementary data (Additional file 2). There were no significant differences in age distribution and postmortem intervals among the disease groups included in this study, as shown in supplementary data (Suppl. Fig. 1).

### Sucrose density gradients

**Fig. 1:**
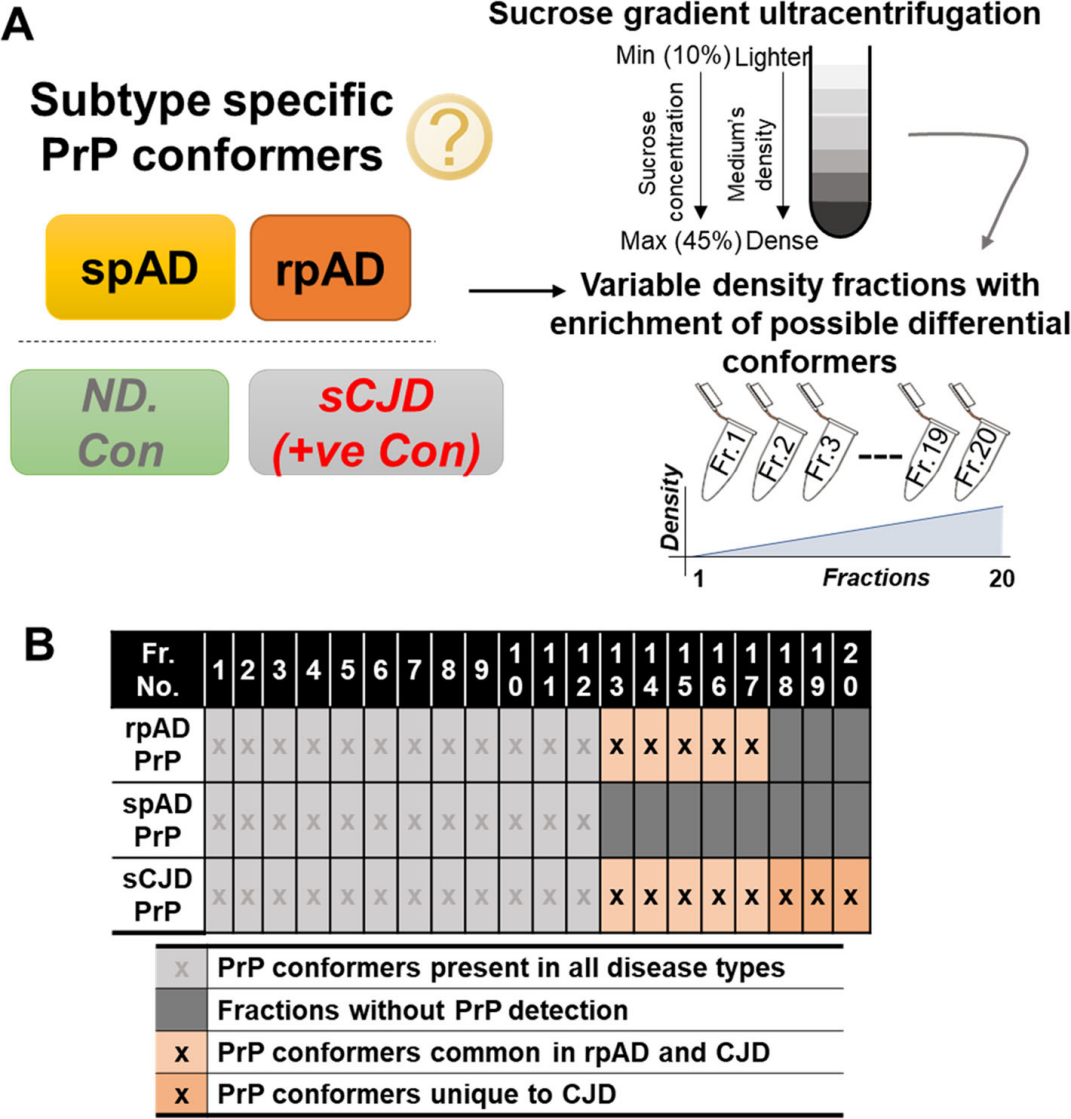
Experimental setup and characterization of disease-specific PrP conformers. A) Scheme of the fractionation of different conformers. Density gradient centrifugation with a 10–45% sucrose step gradient was used to separate the density variants. Centrifugation was carried out at 50,000 rpm and 5 °C. Twenty density fractions were taken from top to bottom (lighter to dense) and used for downstream biochemical assays. B) Profile of high-density PrP (HDP) oligomer occurrence in cortical isolates of rpAD, spAD, sCJD patients (+ve con) and non-demented controls (ND. Con). In contrast to spAD, HDP oligomers were detected in density fractions 12 to 17 in rpAD, thus overlapping with HDPs isolated from sCJD samples

Density gradient fractions were prepared as described previously [8]. Briefly, frontal cortex homogenates (10% w/v in PBS/2% sarkosyl, pH 7.4) were centrifuged at 500 x g for 5 min. Then 400 μL of the supernatant was layered on a 10–45% sucrose gradient prepared in a 13 × 51 mm Beckman thin-wall polyallomer tube by layering serially diluted sucrose solution (10, 15, 20, 25, 30, 35, 40, 45% w/v in PBS/1% N-lauryl sarkosyl, pH 7.4). Ultracentrifugation was carried out at 5 °C, 50,000 rpm for 73 min in an Optima TL 60 ultracentrifuge equipped with a SW-55ti rotor (Beckman Coulter). From each sample, twenty density fractions (200 μL each) were collected from top (lighter) to bottom (denser) fractions separately (Fig. 1a).

#### Preparation of protein and peptide pools from high-density gradient fractions

**Fig. 2:**
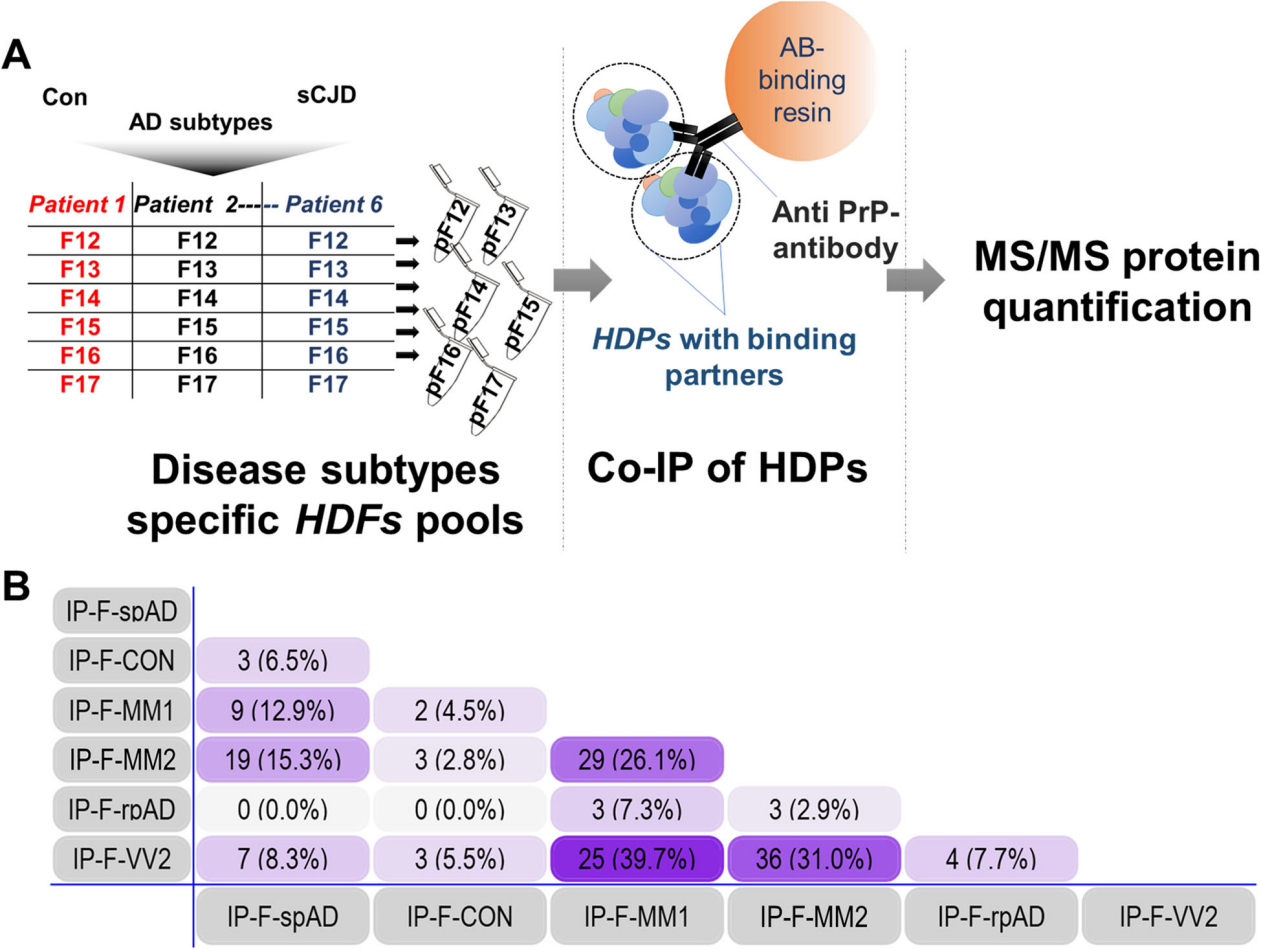
Disease subtype-specific interactors bound to high-density prion protein oligomers. A) The experimental setup for carrying out the co-immunoprecipitation of HDPs and their potential interactors. B) Numerical Venn-diagram showing the overlap of subtype-specific HDP interactors. pF12-pF17: pools of high-density fractions 12–17 from multiple patient samples. IP-F-Con: high-density PrP (HDP) interactors in control HDFs pools from 12 to 17 (collectively), IP-F-spAD: HDP interactors in spAD HDFs pools from 12 to 17, IP-F-rpAD: HDP interactors in rpAD HDFs pools from 12 to 17, IP-F-MM1: HDP interactors in sCJD-MM1 HDFs pools from 12 to 17, IP-F-VV2: HDP interactors in sCJD-VV2 HDFs pools from 12 to 17, IP-F-MM2: HDP interactors in sCJD-MM2 HDFs pools from 12 to 17

For co-immunoprecipitation and subsequent mass spectrometric analysis, equal volumes of corresponding density gradient fractions from the biological replicates (*n* = 6) of each pathological cohort were pooled together (Fig. 2). The pooled high density factions (HDFs) were further subjected for mass spectrometric analysis and co-immunoprecipitation assays.

### Antibodies

All primary and secondary antibodies used for immunoblotting and co-immunofluorescence in this study are listed in Suppl. Tables 1 and 2.

### Co-immunoprecipitation of density fractions

To study unique interactomics signatures of PrP^C^ oligomers in the different density fractions, co-immunoprecipitation was employed using the anti-PrP SAF70 antibody. Phosphatase- and protease-inhibitor cocktails were added before proceeding to the co-immunoprecipitation assay. Co-IP kits (catch and release HT immunoprecipitation kit, Merck) were used to ensure assay homogeneity. Co-immunoprecipitation experiments were carried out in a well-controlled setting with resin-only controls (HDFs were directly incubated with the antibody-binding resin without adding the antibody). The proteins detected in the resin-only controls were considered to be due to unspecific binding to the antibody-binding resin and, when detected, were excluded from the HDF-IP eluates.

### Mass spectrometric analysis

#### Protein/peptide sequence identification by data-dependent acquisition

The HDFs and the Co-IP eluates were separated on 4–12% Bis-Tris gradient gels (NuPAGE Novex Bis-Tris Mini gels, Invitrogen). Following Coomassie staining, the stained areas were excised, diced, and washed in ddH_2_O. Peptide digestion and identification was carried out following protocols described previously [52, 56]. The eluents were analyzed on a Q Exactive hybrid quadrupole/orbitrap mass spectrometer using Excalibur v2.4 software (Thermo Fisher Scientific) and a top10 method in data dependent acquisition mode for analyzing the peptide ions. Raw2MSM v1.17 software (MPI for Biochemistry, Martinsried, Germany) extracted tandem mass spectra and performed database searching. MS/MS spectra were evaluated using Mascot (Matrix Science, London, UK; version 2.4.1) instructed to search the *Homo sapiens* reference proteome (UniProt/SwissProt, revision 02–2017, 92,928 entries) with a 5 ppm precursors mass tolerance and a 0.02 Da mass tolerance for fragments. Each of the Co-IP eluates and HDFs was analyzed twice for MS/MS (two technical duplicates) to reduce data noise. Only peptides identified with a confidence level greater than 95.0% were accepted, and a minimum peptide score of two was required for a peptide identification to be considered as valid.

#### Sequential windowed Acquisition of all Theoretical Fragment ion Mass Spectra (SWATH)-based proteomics

For global proteomic analysis, frontal cortex homogenates (50 μg total protein per sample), of various dementia groups (spAD: *n* = 3, rpAD: n = 3, DLB: *n* = 3, SVD: n = 3, DFTL: n = 3, and rDLB: *n* = 2) and controls (n = 3) were utilized. Two independent MS/MS measurements (technical replicates) were made for each sample to improve the statistical confidence.

To prepare the peptide library, homogenates from each sample were pooled and separated into eight fractions using a reversed phase spin column (Pierce High pH Reversed-Phase Peptide Fractionation Kit, Thermo Fisher Scientific). The separated fractions were then subjected to tryptic digestion as described previously [52, 56]. The protein digests were analyzed on an Eksigent nanoLC425 nanoflow chromatography system associated with a TripleTOF 5600^+^, hybrid triple quadrupole-TOF mass spectrometer equipped with a Nanospray III ion source (Ionspray voltage 2400 V, interface heater temperature 150 °C, and sheath gas setting 12), controlled by Analyst TF 1.7.1 (AB Sciex). The peptides were dissolved in loading buffer (2% acetonitrile (ACN) and 0.1% formic acid (FA) in ddH_2_O) to give a final concentration of 0.3 μg/μL. For each analysis, 1.5 μg of digested protein were concentrated on a precolumn (0.15 mm ID × 20 mm, self-packed, Reprosil-Pur120 C18-AQ 5 μm, Dr. Maisch, Ammerbuch-Entringen, Germany) followed by separation on an analytical RP-C18 column (0.075 mm ID × 250 mm, Reprosil-Pur 120 C18-AQ, 3 μm, Dr. Maisch) with a 100 min linear gradient of 5–35% ACN/0.1% FA at a flow rate of 300 nL/min. Qualitative LC-MS/MS analysis was performed using a Top30 data-dependent acquisition method as described previously [57].

For SWATH analysis of the sample homogenates, MS/MS data were acquired using 100 variable size windows across the 400–1200 *m/z* range. Fragments were produced using rolling collision energy settings for charge state 2+, and fragments acquired over an *m/z* range of 180–1500 for 40 ms per segment. A 250 ms survey scan resulted in an overall cycle time of 4.3 s. Two replicate injections were acquired for each biological sample. Protein identification was achieved using ProteinPilot Software version 5.0 build 4769 (AB Sciex). A total of 152,341 MS/MS spectra were searched against the UniProtKB *Homo sapiens* reference proteome (revision 02–2017, 92,928 entries). A total of 1756 proteins were identified with a false discovery rate of 1%.

#### Co-immunofluorescence analysis

Coronal sections (5 μm thick) for co-immunofluorescence slides were prepared from formic acid-fixed, paraffin-embedded cortex samples from AD patients (spAD: *n* = 5, rpAD: n = 5) and non-demented controls (n = 5) following a protocol described previously [58]. Confocal microscopy was carried out using an SPE laser-scanning microscope (Leica, Germany; 543 and 633 nm helium-neon and 488 nm argon excitation wavelengths). Ten unbiased micrographs were scanned per tissue section from the cortical grey matter area. Individual images were separately analyzed for co-localization using the ImageJ (WCIF plugin) software. Threshold Mander’s overlap coefficient and Pearson’s linear correlation coefficient (*rP*) values were calculated to quantify fluorescence channel correlations and to illustrate the strength and direction of the linear relationship between the two fluorescence channels. One-way ANOVA followed by Tukey**’**s post-hoc test was used to compare the mean values of Threshold Mander’s overlap coefficients.

## Results

### High-density prion protein oligomers (HDPs)

Specific occurrence of HDPs has been reported in brain homogenates of rpAD compared with spAD in our previous work [8]. Density gradient ultracentrifugation was performed using 10–45% step gradients to obtain twenty varying density fractions. Oligomeric prion protein was detected consistently in the isolated fractions 12 to 17 (Fig. 1b) [8]. Due to the unique overlap of properties of the high-density fractions of rpAD and sCJD, the fractions 12 to 17 were further used for downstream interactomics (Fig. 2a).

### Identification of interactors binding to HDPs in brain tissue

The HDPs with the interacting proteins were isolated from the density gradient fractions by immunoprecipitation and were characterized by MS/MS analysis as described above. A total of six interactors were identified, either uniquely present in rpAD-specific HDFs or commonly shared by rpAD and sCJD subtype-specific HDFs, whereas the HDP interactomes of controls and spAD subtypes had no common interactors with rpAD. Intriguingly, some of the HDP interactors from rpAD HDFs were also commonly found as the interactors of HDP conformers from sCJD subtypes. Three HDP interactors, namely mammalian ependymin-related protein 1 (EPDR1), Calcium/calmodulin-dependent protein kinase type II (CaMKII) subunit beta (CAMK2B*)* and CaMKII subunit delta (CAMK2D), were found in Co-IP eluates of rpAD HDFs (IP-F-rpAD) and sCJD-MM1, -MM2 and VV2 subtype fractions, i.e. IP-F-MM1, IP-F-MM2 and IP-F-VV2, respectively. IP-F-VV2 and IP-F-rpAD also shared another common interactor, namely GTP-binding protein Di-Ras2 (DIRA2) and 14–3-3 protein sigma (1433S), whereas GAS2-like protein 2 (G2L2) was observed to uniquely interact with rpAD-specific HDPs (Fig. 2, Table 1). Subtype-unique HDP interactors and the interactors commonly occurring in samples from both spAD and sCJD subtypes are discussed in the supplementary data section (Suppl. Tables 3–7).

**Table 1:** HDP-binding interactors in rpAD high-density fractions identified by mass spectrometry assisted co-immunoprecipitation using anti-PrP antibody

No.	IDs	UniProt Acc. No.	Protein name	Occurrence	Sub-cellular location	Disease Relevance
1	KCC2B	Q13554	Calcium/calmodulin-dependent protein kinase type II subunit beta	**rpAD-F12**, sCJD-MM1-F13, 17, sCJD-MM2-F16, 17, sCJD-VV2-F16, 17	C, Ck, Ce, Sr, Sy	Alzheimer’s disease [59]
2	KCC2D	Q13557	Calcium/calmodulin-dependent protein kinase type II subunit delta	**rpAD-F12**, sCJD-MM1-F13, 17, sCJD-MM2-F16, 17, sCJD-VV2-F16, 17	Cm, Sl	
3	EPDR1	Q9UM22	Mammalian ependymin-related protein 1	**rpAD-F12**, sCJD-MM1-F13–16, sCJD-MM2-F13–16, sCJD-VV2-F12, 14, 15	S	Unknown
4	DIRA2	Q96HU8	GTP-binding protein Di-Ras2	**rpAD-F13**, **15,** sCJD-VV2-F17	Cm	Unknown
5	G2L2	Q8NHY3	GAS2-like protein 2	**rpAD-F16**	C, Ck	Unknown
6	1433S	P31947	14–3-3 protein sigma	**rpAD-F17**, sCJD-VV2-F17	C, Nu, S	Parkinson’s disease [60], Alzheimer’s disease, Creutzfeldt Jakob disease [61], Epilepsy [62]

F12 to F17: HDF-pool 12 to 17. Ce: centrosome, Sy: synapse, Sr: sarcoplasmic reticulum, C: cytoplasm, Ck: cytoskeleton, Nu: nucleus, S: secreted, Cm: cell membrane, Sl: sarcolemma, The localization of proteins and accession number are assigned as in the ExPASy protein database and Uniprot data base, respectively. Disease relevance of the HDP-interacting proteins was identified using Uniprot database search, as well

### Expression of G2L2 and associated proteins

**Fig. 3:**
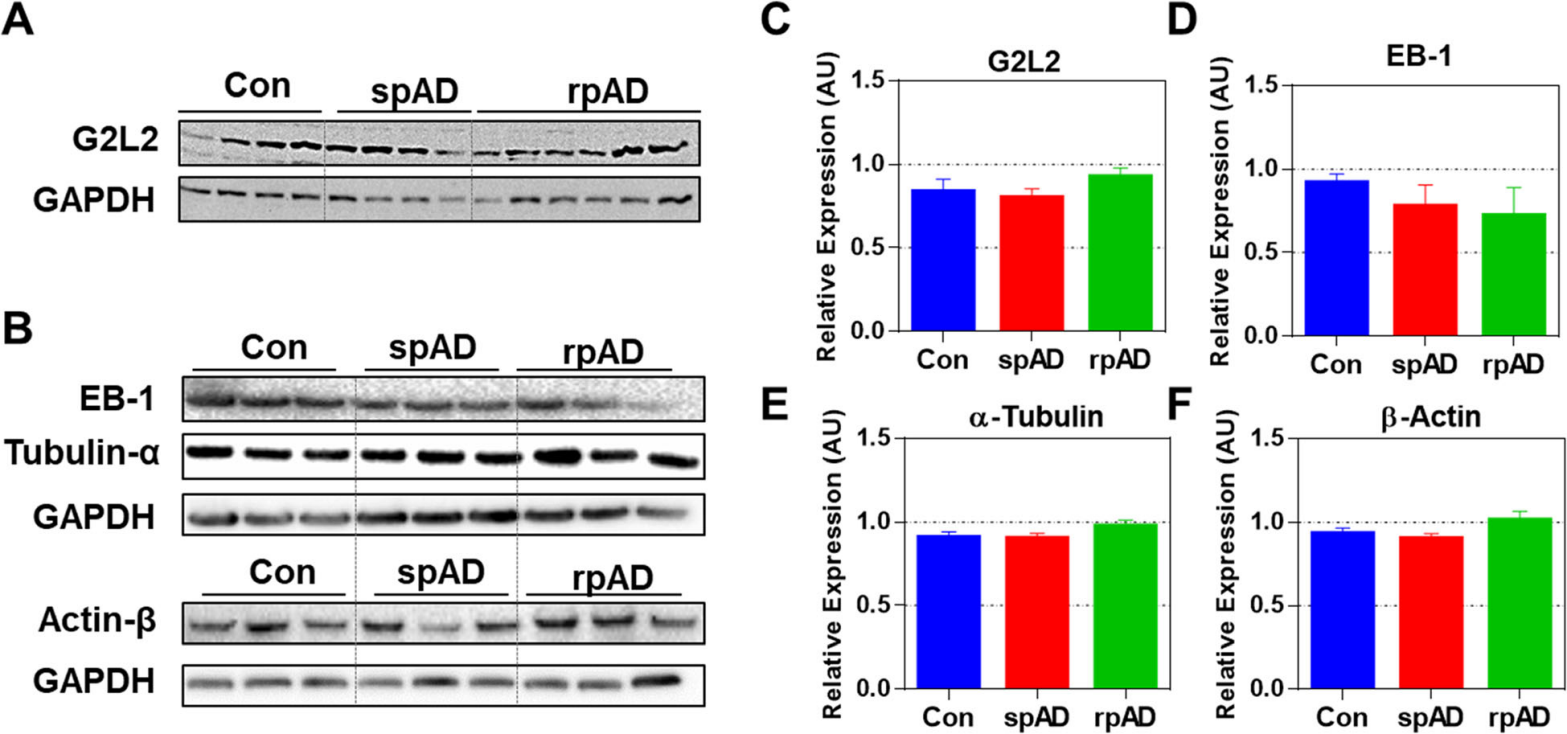
Expression of G2L2 and associated proteins in the frontal cortex of experimental cohorts. A & B) Immunoblots showing the expression levels of G2L2, EB-1, α-tubulin, and β-actin. GAPDH served as loading control. C-F) Densitometric quantification for expression of G2L2, EB-1, α-tubulin, and β-actin in spAD (*n* = 7), rpAD (n = 7), and control (*n* = 6) cases, as assessed in three technical replicates. No significant differences were found for G2L2 and its associated proteins. Statistical significance was calculated with one-way ANOVA followed by Tukey’s post-hoc test

Among the proteins identified as HDP interactors in rpAD, G2L2 was selected for further functional verifications, given its essential role in cytoskeletal integrity, i.e. in actin-mediated microtubule growth, and the possible relevance of the latter for AD pathology. G2L2 is reported for its role in co-alignment of actin filaments and microtubules, where it acts by cross-linking the two structures. This actin/microtubule cross-linking is assisted by the end-binding protein-1 (EB-1) at the plus end of the microtubules [63–65]. In order to study G2L2 in the sample cohorts in more detail, proteins functionally associated with the physiology of G2L2, including EB-1, tubulin and actin, were also studied. Expression of these proteins in frontal cortex homogenates was assessed by western blot analysis. We found no significant differences in the levels of G2L2 between the AD subtypes and controls. Likewise, no significant differences were detected between the groups for EB-1, α-tubulin, and β-actin (Fig. 3).

### Differential co-localization of G2L2 and HDP affects the cytoskeletal integrity in the neurons

#### Neuronal co-localization of G2L2 and HDP affects G2L2/EB-1 binding

**Fig. 4:**
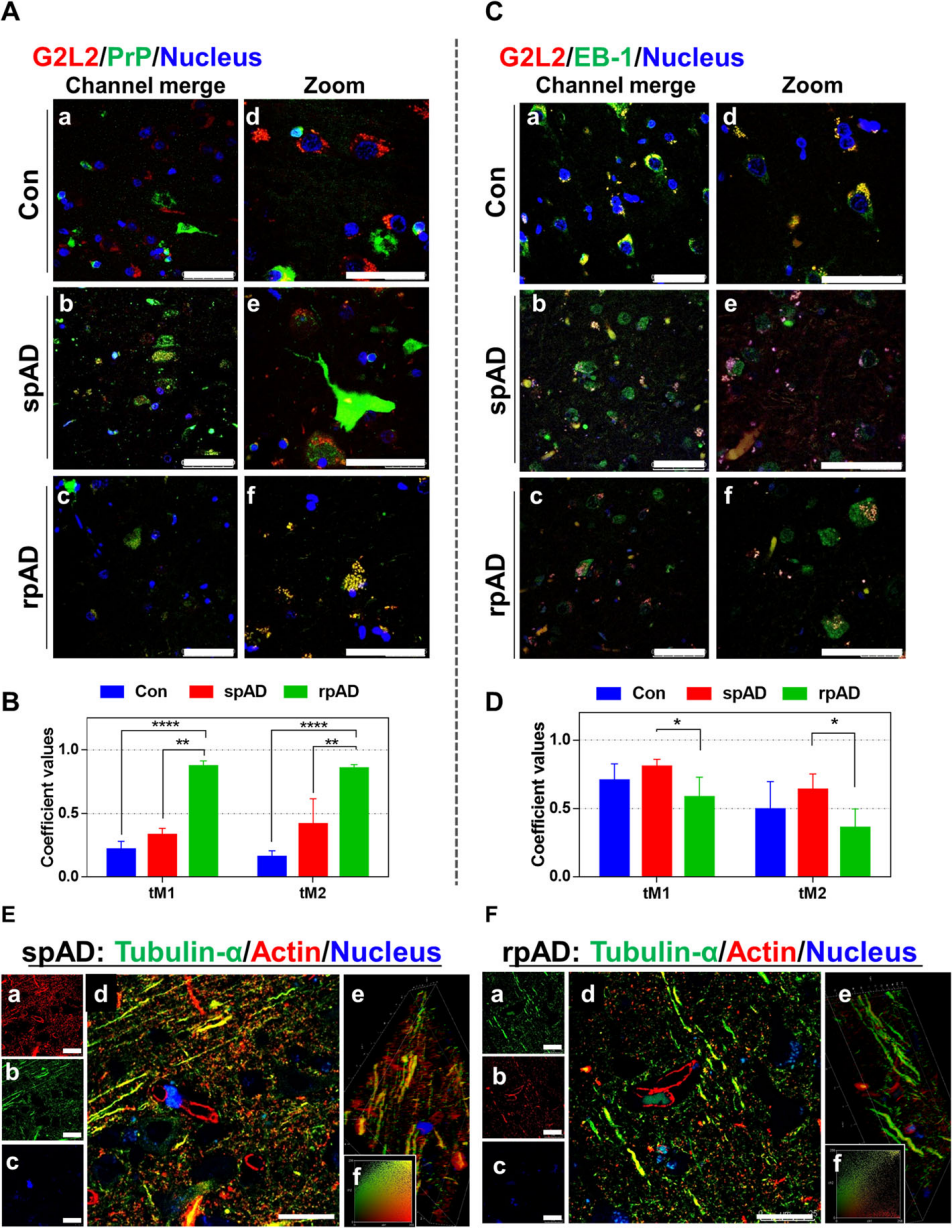
Neuronal co-localization of G2L2 and HDP effects G2L2 / EB-1 binding and cytoskeletal integrity in the rpAD brain cortex. A) Representative photomicrographs (panels Aa-Af) from frontal cortex sections of rpAD, spAD and controls stained with anti-G2L2 and anti-PrP (SAF70) antibodies. Highest co-localization of G2L2 and PrP was observed in frontal cortex sections from rpAD, followed by spAD and controls. B) Threshold Mander’s coefficient values for the overlap of G2L2 channel pixels to PrP channel pixels (tM1) were significantly higher in rpAD than in spAD. The average tM2 (Mander’s coefficient for the overlap of PrP channel pixels to G2L2 channel pixels) was also significantly higher in rpAD than that of spAD. C) Representative frontal cortex sections of rpAD, spAD and controls stained for G2L2 and EB-1 (panels Ca-Cf) show lowest G2L2/EB-1 co-localization in rpAD frontal cortex sections followed by those of spAD and control, respectively. D) Significantly decreased tM1 (G2L2) and tM2 (EB-1) values were seen in rpAD compared with spAD and Con sections. Statistical significance was calculated with one-way ANOVA followed by Tukey’s post-hoc test. *p < 0.05; **p < 0.005; ***p < 0.001*.* E&F) Representative micrographs of spAD (E) and rpAD (F) sections stained using anti-α-tubulin and anti-β-actin antibodies are shown. Panels Ea-Ec and Fa-Fc show the single channel images. Panels Ed and Fd show the channel merges. Panels Ee and Fe correspond to the 3D reconstructions from z-stacks of spAD and rpAD, respectively. Insets Ef and Ff show representative IC plots. Relatively stronger actin/tubulin co-localization was observed in spAD compared with rpAD patients. Confocal images were scanned from the frontal cortex sections of spAD (*n* = 5), rpAD (n = 5) and controls (n = 5). Scale bars in panels A&C = 50 μm; in panels E&F = 25 μm

Immunohistological observations were made in the grey matter areas of the frontal cortex. A certain subtype-specific trend was observed in G2L2/PrP co-localization. The highest level of co-localization between PrP^C^ and G2L2 was observed in rpAD followed by spAD samples (Fig. 4a). Threshold Mander’s overlap coefficients values have been used to assess the co-localization of red and green channels in the microscopic images. Where tM1 represents the overlaps of pixels from the red channel (G2L2) on those of the green channel (PrP), and tM2-overlaps of pixels from the green channel (PrP) on those of the red channel (G2L2). Mander’s overlap coefficient values reveal the highest G2L2-PrP^C^ co-localization in rpAD followed by spAD and controls (Fig. 4b). However, a significant decrease in G2L2 and EB-1 co-localization was observed in rpAD compared with spAD and controls (Fig. 4c). The differences in the Mander’s overlap coefficient values (Fig. 4d) also represent the lowest levels of co-localization in rpAD followed by a higher overlap in spAD and the highest in the controls.

#### Disturbance in G2L2/EB1 co-localization is associated with loss of β-actin/α-tubulin integration

Actin-tubulin co-alignment was also studied using confocal laser scanning microscopy. Frontal cortex sections (5 μm thick) were stained for α-tubulin and β-actin. Confocal z-sections were scanned and later used to construct three-dimensional images. A more pronounced actin-tubulin co-localization was observed in the axonal processes of gray matter neurons of the spAD samples in comparison with rpAD (Fig. 4e, panel d and Fig. 4f, panel d). Likewise, correlation plots prepared for the actin and tubulin channels revealed a significantly greater overlap between the channel intensities (Fig. 4e, panel f and Fig. 4f, panel f). Three-dimensional reconstructions of the z-sections also showed longer stretches of filaments with actin-tubulin co-localization in spAD compared with rpAD, with higher actin and tubulin channel overlap (Fig. 4e, panel e and Fig. 4f, panel e).

### Proteomics of HDFs also indicate higher degree of cytoskeletal disintegration in rpAD

**Fig. 5:**
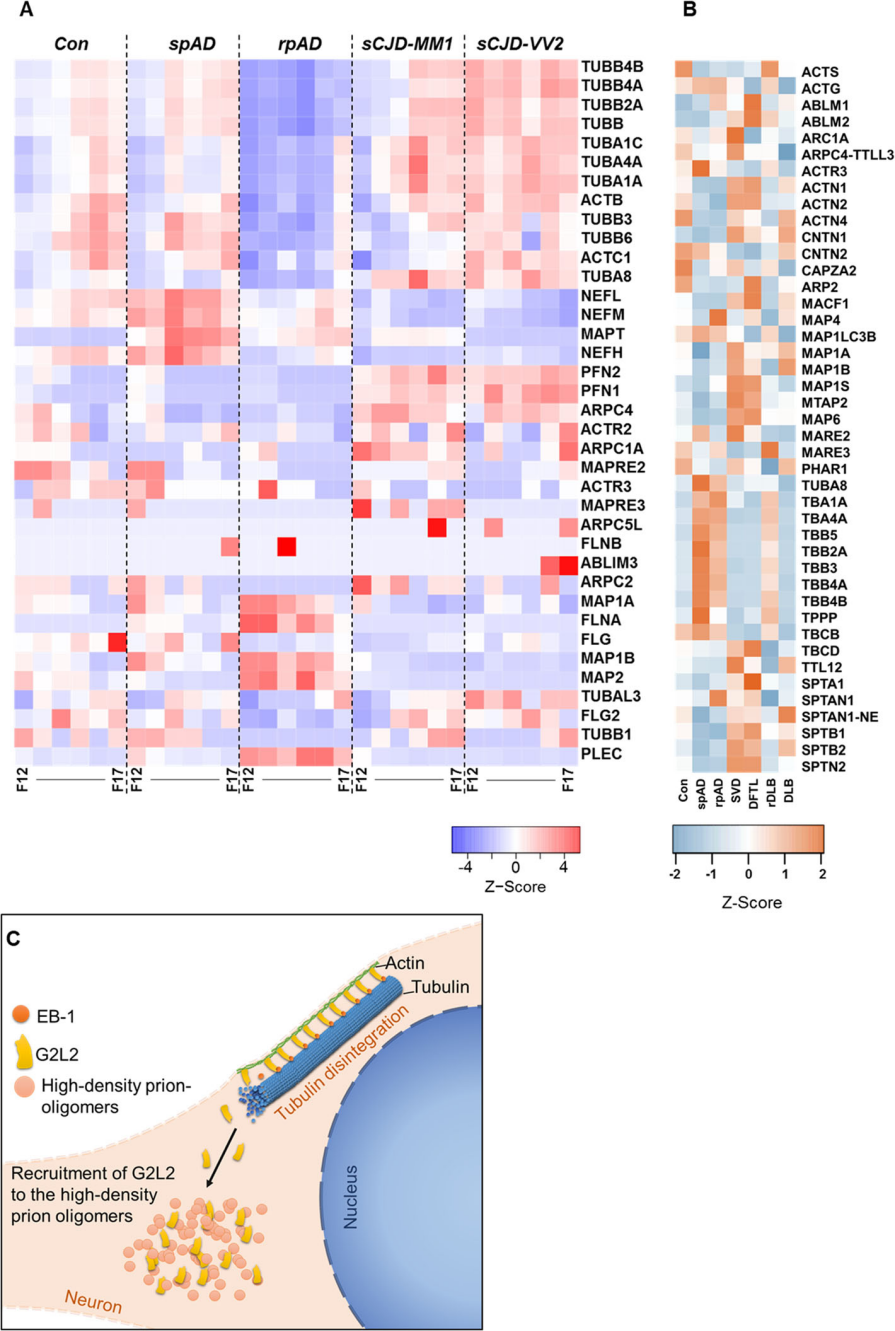
Variations in cytoskeletal proteins in high-density fractions. A) The relative abundance of cytoskeletal proteins in HDFs detected by high-resolution MS/MS analysis is represented as z-score. Significant reduction of cytoskeletal proteins was seen in rpAD-specific HDFs (F12–F17: HDF pool 12 to 17). B) Differences in expression of cytoskeletal and associative proteins as determined by the SWATH-MS. Heatmaps represent the relative protein expression indicated as z-scores for spAD (*n* = 3), rpAD (n = 3), DLB (n = 3), rDLB (*n* = 2), D-FTL (n = 3), SVD (n = 3), and controls (n = 3). C) Model showing the recruitment of G2L2 to high-density prion-oligomers. The recruitment of G2L2 towards HDPs results in the loss of its binding to EB-1, affecting the actin-guided microtubule (MT) integrity

MS/MS analysis of HDF pools was carried out and the observed proteomic variations (unique peptide counts) were compared with the baseline global proteomic data sets obtained by SWATH-MS to recognize variations in cytoskeletal proteins. Levels of tubulin subunits were observed to be significantly reduced in HDFs from rpAD patients compared with HDFs from other groups. A significant decrease was also seen in the levels of certain neurofilament subunits, actin and actin-binding proteins in rpAD HDFs. Conversely, significantly higher levels of microtubule-associated proteins (MAP 1A, MAP 1B, and MAP 2), flaming (FLNA), filaggrin (FLG) and plectin (PLEC) were observed in rpAD HDFs compared with sCJD-specific HDFs. Interestingly, the levels of the microtubule-associated protein tau (MAPT) were found to be lower in rpAD-specific HDFs compared with spAD-specific HDFs, although higher than the HDFs from Con and sCJD subtypes (Fig. 5a, Additional file 4). Among the cytoskeletal proteins from the global proteome data, the expression of actin G (ACTG) was found to be significantly increased in rpAD compared with controls, whereas no significant differences were observed between the spAD and rpAD. Expression of ACTG was significantly decreased in SVD and DFTL compared with rpAD. Contactin 1 (CNTN1) expression was also found to be significantly higher in SVD and DFTL than in rpAD. Expression of tubulin alpha 1a (TBA1A) was observed to be significantly higher in rpAD than in other rapid dementia samples, i.e. DFTL, SVD, and r-DLB. Likewise, expression of TBA4A was differentially higher in rpAD compared with controls, DLB, DFTL, and SVD (Fig. 5b, Additional file 3).

## Discussion

Multiple PrP conformers have previously been described in association with transmissible spongiform prion diseases in animals and humans [66, 67]. The presence of high-density PrP conformers (HDPs) associated specifically with rpAD have recently been identified by our group [8]. Here we aimed to deepen our understanding of these findings by isolating HDP complexes, identifying HDP interactors, and by downstream proteomic analyses.

Diverse sets of prion protein interactors were identified from subtype-specific HDFs. In our study, we identified fewer rpAD-specific HDP interactors compared with sCJD. As reported previously, sCJD tissues exhibit a wider spectrum of PrP/PrP^Sc^ oligomers [66, 68, 69] compared with rpAD [8]. A greater diversity of PrP/PrP^Sc^ oligomers in sCJD pathology can be a potential reason for the relatively diverse interactome found for sCJD PrP oligomers. There was no overlap of HDP-interactors of rpAD samples with either spAD or control patient tissue, reaffirming the subtype-specific occurrence of certain PrP species in rpAD. Interactors common for the rpAD and sCJD datasets point towards the presence of common PrP oligomers present in both disease entities. Proteins interacting with the HDPs in rpAD included CaMKII subunits (KCC2B, KCC2D), EPDR1, DIRA2, G2L2, and 1433S. All of these interactors, except for the G2L2 protein, were part of the HDP-interactomes in both rpAD and sCJD. CaMKII, besides regulating Ca^2+^ in neurons, is also responsible for reorganizing actin into bundles in the development of dendritic spines [70]. GTP-binding Di-Ras2 is a Ras GTPase known for its involvement in determining cell morphogenesis. G2L2 has been reported to play a role in actin-tubulin communication and connectivity [71]. Protein 14–3-3 sigma is a protein with many known interactions and is involved in p53, protein kinase C, and AKT/mTOR signaling [72]. Strong evidence had been presented in the literature for the involvement of 14–3-3 isoforms in various neurodegenerative disease, particularly in prion dementias and Alzheimer’s disease, where 14–3-3 isoforms are found associated with PrP^Sc^ and Aβ plaques and serve as biomarker [73, 74]. Physiological outcomes of novel interactions of G2L2 with the PrP oligomers are discussed in the following. Actin and tubulin interlinking and communication are necessary for the proper functioning of cellular transport systems, morphogenesis, repair and many other related functions in the cells. This link is mainly controlled by spectraplakins via their ability to connect actin and tubulin filaments [40]. Likewise, the family of growth arrest-specific (GAS) proteins and GAS-like (GASL) proteins plays a role in linking actin and tubulin filaments [71]. Mutations in the G2L2 protein were reported to increase the risk of developing Alzheimer’s disease [75]. Stroud and coworkers [63] have provided a comprehensive experimental account of the GAS and GASL and proposed a model in which the GASL proteins G2L1 and G2L2 control microtubule stability via attachment to end-binding protein-1 (EB-1). The interaction of G2L2 and EB-1 with tubulin is also reported to account for microtubular stability [63]. In our study, we found a negative correlation between HDP-G2L2 interaction and G2L2 and EB-1 binding in the rpAD cohort. This disturbance in the G2L2/EB-1 system may lead to a malfunction of actin-assisted microtubule growth in neurons. The shortening of the actin-tubulin co-localization fibers that is specifically observed in the frontal cortex of rpAD patients can be interpreted as a consequence of the disturbances in the G2L2/EB-1/tubulin system. Figure 5c illustrates a possible mechanism of how HDPs interact with the cytoskeletal system. Proteomic data of HDFs also show more extensive cytoskeletal disintegration in rpAD than in spAD and control patients. Decreased levels of tubulin in rpAD HDFs are indicative of greater damage to the cytoskeletal system, fitting to what has previously been described [76]. The decrease in tubulin integrity is a sign of various pathological cascades associated with rpAD that differ from those associated to spAD. PrP^C^ was reported to inhibit microtubule synthesis by its direct interaction with tubulin [53, 54]. The PrP oligomers uniquely identified in rpAD may be associated with tubulin-sequestering that results in a greater degree of microtubule damage. Low tubulin HDF levels in rpAD are accompanied by a decrease in the MAPT levels as well as higher levels of MAP 1 and MAP 2. A previous report by Ba et al. (2016), with rapid progression defined on the basis of cognitive decline compared to that of survival time in our study, showed a significant decrease in the p-tau/tau ratio in cerebrospinal fluid (CSF) of rpAD patients (*n* = 55) compared with that in spAD patients (*n* = 257) [17]. As the p-tau molecules change their conformation and undergo oligomerization, ultimately leading to the formation of tau tangles [77], the aforementioned discrepancy in CSF p-tau/tau levels in rpAD patients indicates that the rpAD cohort will show a decrease in MAPT oligomers. The cortical expression of MAP 1 and MAP 2 however does not differ between rpAD and spAD as seen in the global proteomic data. Elevated HDF MAPs levels can result from their self-interaction [78] or from a stronger interaction with tubulin, actin and other regulatory factors, such as kinases, as previously reported [78–83]. MAP sequestering also correlates with the loss of microtubule integrity in the rpAD cortices. Together with the malfunctioning in the microtubule system, neurons suffer from a variety of other degenerative events. Considering the size and complexity of a neuron, a robust intracellular transport system is obviously necessary to maintain the critical connections between the cell body and its distant neural processes for supplying the various types of neuronal cargo, such as organelles, vesicles, cell signaling molecules, RNA molecules, neurotransmitters, receptors, and adhesion molecules [36, 37]. The actin-guided microtubule growth also plays a critical role in the structural stability of neurons including kinesin-based axon differentiation and polarization [37, 84], MAP-assisted axon growth [85], and finally the morphodynamics of dendritic spines [36, 37]. The higher levels of the cytoskeletal proteins in sCJD HDFs indicate that a different set of mechanisms is involved in CJD pathology. Interaction between tubulin and PrP has been reported by many previous studies [53, 54]. We argue that the higher levels of cytoskeletal proteins (tubulin isoforms and MAPs) found in the sCJD high-density fractions in our study are correlated to the relatively higher levels of HDPs with which they interact.

## Conclusion

The results suggest an involvement of high-density PrP oligomers in the cytoskeletal damage of the frontal cortex specific to rpAD, as indicated by confocal microscopy and proteomic profiling. The proposed competitive binding of HDPs to G2L2 resulted in the interruption of G2L2/EB-1/Tubulin interaction, which lead to a greater extent of disintegration and damage to the cytoskeletal system. These rpAD-specific cytoskeletal alterations  can contribute in the accelerated disease progression of rpAD patients.

## Supplementary Information

**Additional file 1 **The document file contains supplementary figures and tables. Suppl. Fig. 1**:** Summary of frontal cortex cohorts used in current study. Further clinical features and neuropathological details of the cohort are given in the Additional file 2. Suppl. Fig. 2 Sample cohorts used in the study. A) Comparison of ages of the diverse pathological cohorts used in the study. B) Graph presents a comparison of post-mortem intervals to the time of autopsies. Suppl. Table 1: List of primary antibodies and their applications in the current study. Suppl. Table 2: List of secondary antibodies and their applications in current study. Suppl. Table 3: High-density PrP (HDP) interactors commonly found in the HDFs of all sCJD subtypes. Suppl. Table 4: High-density PrP (HDP) interactors commonly detected between the high-density fractions of sCJD-MM2 and sCJD-VV2 subtypes.

**Additional file 2.** The spreadsheet contains the details of the sample cohort used in the study including the clinical data, the neuropathological profiling, and the experiment designing.

**Additional file 3.** The spreadsheet contains the differential expression of cytoskeletal and cytoskeletal-associated proteins from the frontal cortex tissue lysates of Con, spAD, rpAD, SVD, DFTL, rDLB and DLB patients assessed using the SWATH-MS. Data shown are the normalized SWATH averages and the corresponding SEM values. The spreadsheet also contains the results of the post-hoc intergroup analyses for the differential expression SWATH values of these proteins.

**Additional file 4.** The spreadsheet gives the average peptide counts and SEM of the cytoskeletal and cytoskeletal-associated proteins from the HDF pools from frontal cortex lysates of control brains and those other neurodegenerative samples described in the study. It also contains the results of the post-hoc intergroup analyses of these peptide counts.

**Additional file 5.** The spreadsheet contains the raw global proteomics data (SWATH-MS values) of Con, spAD, rpAD, DLB, rDLB, DFTL, and SVD. Subsequent normalized data along with the results of pairwise statistical testing (Welch’s t-test).

**Additional file 6.** The spreadsheet contains the raw proteomics data (unique peptide counts data) of HDFs, and subsequent normalized data along with the results of pairwise statistical testing (Welch’s t-test).

**Additional file 7.** The additional file contains spreadsheets with averaged normalized mass spectrometric data after removal of candidates found in beads only controls (unique peptide counts), for the interactors of high-density prion protein oligomers in cerebral cortices from controls and neurodegenerative cases included in the study.

## Data Availability

All data generated or analyzed during this study are included in this published article [and its supplementary information files].

## Abbreviations

ACN: acetonitrile
AD: Alzheimer’s disease
ADAM: A disintegrin- and metalloproteinase
AgNO_3_: silver nitrate
AKT/PKB: protein kinase B
APOE: apolipoprotein E
APP: amyloid precursor protein
Aβ: amyloid-β
CaMKII: calcium/calmodulin-dependent protein kinase type II
cAMP: cyclic adenosine monophosphate
Co-IP: co-immunoprecipitation
CSF: cerebrospinal fluid
ddH2O: double distilled water
DFTL: dementia with fronto-temporal lobar degeneration
DLB: dementia with Lewy bodies
DSG2: desmoglein 2
DTT: dithiothreitol
EDTA: ethylenediaminetetraacetic acid
Erk: extracellular signal–regulated kinases
ESI: electrospray ionization
FAD: familial AD
G-CSF: granulocyte-colony stimulating factor
GSK3: glycogen synthase kinase 3-β
HDFs: high density fractions
HDP: high density prions
HMW: high molecular weight
IAA: iodoacetamide
IgG: immunoglobulin G
IL-13: interleukin 13
IL-6: interleukin 6
JNK: c-jun N-terminal kinases
KCC2: calcium/calmodulin-dependent protein kinase type II
kDa: kilodalton
LMW: low molecular weight
LTP: long-term potentiation
MAPKs: mitogen-activated protein kinases
MCI: mild Cognitive Impairment
MCP*-*1: monocyte chemoattractant protein-1
MMSE: Mini-Mental State Examination
MS/MS: mass spectrometery
MYLK: myosin light chain kinase
Na_2_S_2_O_3_: sodium thiosulfate
NaCl: sodium chloride
NFkB: nuclear factor kappa-light-chain-enhancer of activated B cells
NFTs: neurofibrillary tangles
NH_4_HCO_3_: sodium bicarbonate
NP-40: nonidet P-40
NCAM: neuronal cell adhesion molecule
p38: mitogen-activated protein kinases P38
PBS: phosphate buffered saline
PKA: protein kinase A
PKC: protein kinase C
PLD3: phospholipase D3
PRNP: prion protein coding gene
PrP: prion protein
PrP^C^: cellular prion protein
PrP^Sc^: scrapie form of prion protein
*PSEN1*: presenilin 1
*PSEN2*: presenilin 2
p-tau: phosphorylated tau
Q-TOF: quadrupole time of flight
rDLB: rapidly progressive dementia with Lewy bodies
recPrP: recombinant PrP
RFU: relative fluorescence units
*rP*: Pearson’s linear correlation coefficient
rpAD: rapidly progressive Alzheimer’s disease
SAPK: stress-activated phosphokinases
sAPPα: shed-APP
sCJD: sporadic Creutzfeldt-Jakob disease
SDS: sodium dodecyl sulfate
SDS-PAGE: sodium dodecyl sulfate- polyacrylamide gel electrophoresis
spAD: sporadic Alzheimer’s disease
*SRC*: proto-oncogene tyrosine-protein kinase
SVD: small vessel disease
SWATH-MS: sequential window acquisition of all theoretical mass spectra- mass spectrometry
Tau: tubulin associated unit
TBS: Tris buffered saline
TBST: TBS with 0.1% Tween
TEMED: tetramethylethylenediamine
Th-T: thioflavin-T
tM: threshold Mander’s coefficient
TNF: tumor necrosis factor
TREM2: triggering receptor expressed on myeloid cells 2
Tris: tris (hydroxymethyl)aminomethane
TSEs: transmissible spongiform encephalopathies
