# Supplementary material for: Prion protein oligomers cause neuronal cytoskeletal damage in rapidly progressive Alzheimer’s disease
Source: Mol Neurodegener. 2021 Feb 22;16:11. doi: 10.1186/s13024-021-00422-x (PMC7898440; doi:10.1186/s13024-021-00422-x)
Supplement: Supplementary file 1 — Additional file 1 The document file contains supplementary figures and tables. Suppl. Fig. 1: Summary of frontal cortex cohorts used in current study. Further clinical features and neuropathological details of the cohort are given in the Additional file 2. Suppl. Fig. 2 Sample cohorts used in the study. A) Comparison of ages of the diverse pathological cohorts used in the study. B) Graph presents a comparison of post-mortem intervals to the time of autopsies. Suppl. Table 1: List of primary antibodies and their applications in the current study. Suppl. Table 2: List of secondary antibodies and their applications in current study. Suppl. Table 3: High-density PrP (HDP) interactors commonly found in the HDFs of all sCJD subtypes. Suppl. Table 4: High-density PrP (HDP) interactors commonly detected between the high-density fractions of sCJD-MM2 and sCJD-VV2 subtypes. [file 13024_2021_422_MOESM1_ESM.docx]

# SUPPLEMENTARY figures and tables

**
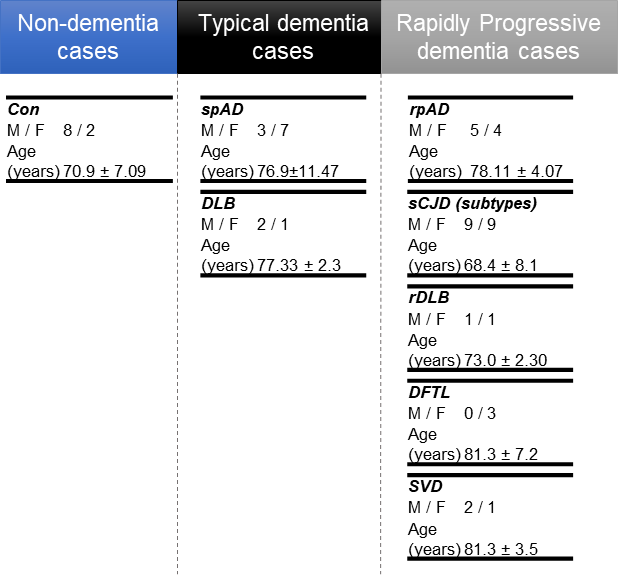
**

Suppl. figure 1: Summary of frontal cortex cohorts used in current study. Further clinical features and neuropathological details of the cohort are given in the Additional file 2.


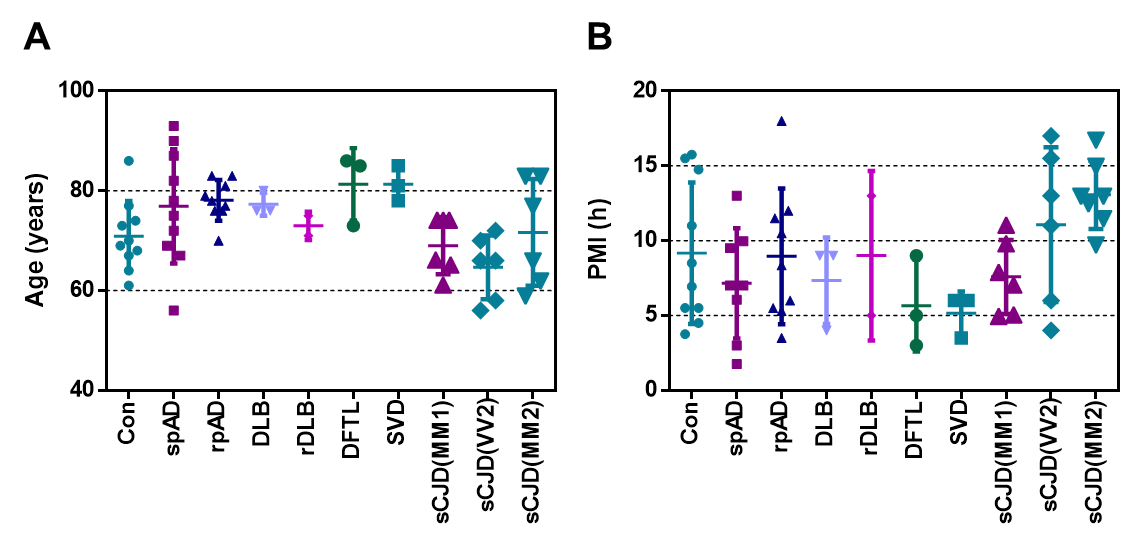


Suppl. figure 2: Sample cohorts used in the study A) Comparison of ages of the diverse pathological groups used in the study. B) Graph presents a comparison of post-mortem intervals to the time of autopsies.

Suppl. table 1 : List of primary antibodies and their applications in the current study

| **Primary Antibody** | **Origin** | **Dilution (IB/IP)** | **Dilution (IF)** | **Company/ Cat. No.** |
| --- | --- | --- | --- | --- |
| SAF 70 (anti PrP antibody) | Mouse IgG2b | 1:1000/1:100 | 1:100 | SPIbio / A03206 |
| Actin-beta | Mouse IgM | 1:10000 | 1:100 | Sigma / A5441 |
| Zinc Alpha 2 Glycoprotein | Mouse IgG1 | 1:1000/ 1:100 | 1:100 | Abcam / ab117275 |
| GAPDH | Mouse IgM | 1:10000 | - | Sigma / G8795 |
| G2L2 | Rabbit IgG | 1:1000 | 1:100 | Abcam / ab170275 |
| EB-1 | Rabbit IgG | 1:1000 | 1:100 | Invitrogen / PA5-25913 |
| Tubulin-alpha | Mouse IgG | 1:1000 | 1:100 | Santacruz biotech. / sc-58667 |

Suppl. table 2: List of secondary antibodies and their applications in current study

| **Secondary antibody** | **Origin** | **Dilution (IB)** | **Dilution (IF)** | **Company/ Cat. No.** |
| --- | --- | --- | --- | --- |
| α-Mouse-HRP | Goat | 1:10000 | - | JacksonIR Lab / 115-035-062 |
| α-Rabbit-HRP | Goat | 1:10000 | - | JacksonIR Lab / 111-035-144 |
| α-Goat-HRP | Goat | - | 1:200 | JacksonIR Lab / 705-035-003 |
| α-Mouse-A488 | Goat | - | 1:200 | Thermo Fischer Sci. / A32723 |
| α-Rabbit-A488 | Goat | - | 1:200 | Thermo Fischer Sci. / A-11034 |
| α-Mouse-A546 | Goat | - | 1:200 | Thermo Fischer Sci. / A-11003 |
| α-Rabbit-A546 | Goat | - | 1:200 | Thermo Fischer Sci. / A-11010 |

**High density prion protein interactors in spAD and sCJD**

Nine common interactors between CJD-MM1 and spAD HDFs, likewise, we could also identify three interactors common to the controls and spAD HDFs including cathepsin D (CTSD), catenin beta-1 (CTNNB1) and protein piccolo (PCLO). However, as HDPs are not reported previously for the Con and spAD, the HDP interacting proteins could be detected in Co-IP eluates from spAD and Con HDFs because of unspecific binding to Dynabeads. Nineteen common interactors for CJD-MM2 HDPs and seven from that of sCJD-VV2 were found in spAD-HDFs as well. The number of HDP-interactors for the sCJD-subtypes was higher compared to that of controls, spAD and rpAD and the degree of intergroup overlap between the HDP-interactors from sCJD subtypes was also the highest, presumably due to pathological similarities among the prion strains (**additional file 7**). Aldolase c was commonly identified between HDPs of spAD and VV2. Catenin beta-1 and aconitase hydratase were commonly present between the spAD, VV2 and MM2 HDFs. Three proteins, namely protein piccolo, cathepsin D and triosephosphate isomerase were found commonly interacting to HDPs between the HDFs of spAD, CJD-MM1, CJD-VV2 and CJD-MM2 (**additional file 7**).

Three proteins including calmodulin-like protein 5, endoplasmin and malate dehydrogenase, mitochondrial, were found in the HDFs of spAD, CJD-MM1 and CJD-MM2. Three HDP-interactors, antileukoproteinase, amyloid precursor proteins and 14-3-3E were found in the HDFs from sCJD-MM1 and sCJD-MM2 subtypes (Suppl. figure 3). The high density PrP interactors commonly expressed in all sCJD-subtypes are listed below along-with their disease relevance, and corresponding HDFs (Suppl. table 3).

Suppl. table 3: High-density PrP (HDP) interactors commonly found in the HDFs of all sCJD subtypes.

| **Gene IDs** | **Uniprot Acc. No.** | **Identified Proteins** | **Subcellular location** | **Prion protein-interaction** | **Involvement in disease** | **HDFs-Occurrence** |
| --- | --- | --- | --- | --- | --- | --- |
| **TPP1** | O14773 | Tripeptidyl-peptidase 1 | Ly | Novel | Ceroid lipofuscinosis, neuronal, 2 [38] | sCJD-MM1:F12 toF14,  sCJD-MM2:F12 toF14, F16, F17  sCJD-VV2:F14 |
| **FRIH** | P02794 | Ferritin heavy chain |  | Known [25] | Creutzfeldt-Jakob-disease [25]5 | sCJD-MM1:F12 toF14,  sCJD-MM2:F12, F13,  sCJD-VV2:F12, F13 |
| **EPDR1** | Q9UM22 | Mammalian ependymin-related protein 1 | S | Novel |  | sCJD-MM1:F13 to F16,  sCJD-MM2:F13 toF16, F17,  sCJD-VV2:F12, F14, F15 |
| **PPT1** | P50897 | Palmitoyl-protein thioesterase 1 | Ly | Novel | Ceroid lipofuscinosis, neuronal, 1 [43] | sCJD-MM1:F13 toF16, sCJD-MM2:F12 toF15,  sCJD-VV2:F12 |
| **SYN1** | P17600 | Synapsin-1 | Cj, Sy. Ga | Known [39] | Epilepsy X-linked, with variable learning disabilities and behavior disorders [38] | sCJD-MM1:F12, F16, F17,  sCJD-MM2:F13, F14, F16, F17,  sCJD-VV2:F14 to F17 |
| **SYN2** | Q92777 | Synapsin-2 | Cj, Sy. | Novel | Schizophrenia [38] | sCJD-MM1:F16, F17  sCJD-MM2:F14, F16, F17  sCJD-VV2:F15 toF17 |
| **PACN1** | Q9BY11 | Protein kinase C and casein kinase substrate in neurons protein 1 | Cy, Cp, Cj, Sy, Syo, Cp, | Novel |  | sCJD-MM1:F17  sCJD-MM2:F16, F17,  sCJD-VV2:F15, F17 |
| **COF1** | P23528 | Cofilin-1 | Nu, Cy, Ck, Cp | Known [7] | Creutzfeldt-Jakob-disease [18] | sCJD-MM1:F16, F17  sCJD-MM2:F12, F16, F17,  sCJD-VV2:F16, F17 |
| **CRYM** | Q14894 | Ketimine reductase mu-crystallin | Cy | Novel | Autosomal dominant, 40 deafness [19] | sCJD-MM1:F17,  sCJD-MM2:F16, F17,  sCJD-VV2:F16, F17 |
| **NSF** | P46459 | Vesicle-fusing ATPase | Cy | Novel |  | sCJD-MM1:F17,  sCJD-MM2:F16, F17,  sCJD-VV2:F16, F17 |
| **COF2** | Q9Y281 | Cofilin-2 | Nu matrix cy, Ck | Novel |  | sCJD-MM1:F16, F17,  sCJD-MM2:F12, F16, F17,  sCJD-VV2:F15, F17 |
| **IDH3A** | P50213 | Isocitrate dehydrogenase [NAD] subunit alpha, mitochondrial | Mc | Novel | Alzheimer’s disease [29] | sCJD-MM1:F17  sCJD-MM2:F12, F16, F17  sCJD-VV2:F16, F17 |
| **PROF2** | P35080 | Profilin-2 | Cy, Ck. | Novel | Creutzfeldt-Jakob-disease [30] | sCJD-MM1:F17,  sCJD-MM2:F17,  sCJD-VV2:F16, F17 |
| **NFL** | P07196 | Neurofilament light polypeptide (NF-L) |  | Novel | Prion diseases [33], familial Alzheimer’s disease [34] | sCJD-MM1:F17  sCJD-MM2:F17,  sCJD-VV2:F16, 17 |
| **KCC2G** | Q13555 | Calcium/calmodulin-dependent protein kinase type II subunit gamma | Sr membrane | Novel | Alzheimer’s disease [29] | sCJD-MM1:F13, F17  sCJD-MM2:F16, F17  sCJD-VV2:F16 |
| **GRP75** | P38646 | Stress-70 protein, mitochondrial | Mc, Nu, nucleolus | Novel |  | sCJD-MM1:F17,  sCJD-MM2:F12, F17  sCJD-VV2:F16 |
| **TPPP** | O94811 | Tubulin polymerization-promoting protein | Cy, Ck. Nu. Localizes to glial Lewy bodies | Novel | Known [38] | sCJD-MM1:F17,  sCJD-MM2:F17,  sCJD-VV2:F16 |

F12 to F17: HDF pool-12 to 17. Ce: centrosome, Sy: Synapse, Sr: sarcoplasmic reticulum, C: Cytoplasm, Ck: cytoskeleton, Nu: Nucleus, S: Secreted, Cm: Cell membrane, Sl: Sarcolemma, Ly: Lysosomes, Mc: Mitochondrion, Syo: Synaptosome, Cj: Cell junction, C V: cytoplasmic vesicles, Ga: Golgi apparatus, Pm: phagosome membrane, Px: peroxisome, Em: Endosome membrane, Cp: Cell projection, Gc: growth cone, Ms: Melanosome, Er: Endoplasmic reticulum and La: Lipid-anchor. The localization of proteins and accession number are assigned as in ExPASy protein database and Uniprot data base respectively. Relevance with AD, prion and PrP ligand were established by Uniprot database search as well.

Likewise, eleven proteins were uniquely identified in two sCJD subtype (i.e., MV and VV2)-specific HDFs. Respective reported PrP interaction, disease involvement and specific occurrence in subtype specific HDFs are enlisted in Suppl. table 4.

Suppl. table 4: High-density PrP (HDP) interactors commonly detected between the high-density fractions of sCJD-MM2 and sCJD-VV2 subtypes.

| **No.** | **Gene IDs** | **Entry** | **Protein names** | **Subcellular location** | **PrP-interaction** | **Involvement in disease** | **Occurrence in sub-type specific fractions** |
| --- | --- | --- | --- | --- | --- | --- | --- |
| 1 | DEST | P60981 | Destrin (Actin-depolymerizing factor) |  | Novel |  | sCJD-MM2:F12,  sCJD-VV2:F17 |
| 2 | COR1A | P31146 | Coronin-1A | C, Ck, C, C V, Pm | Novel |  | sCJD-MM2:F12,  sCJD-VV2:F16, F17 |
| 3 | SEPT3 | Q9UH03 | Neuronal-specific septin-3 | C, Ck, Cj, Sy | Novel |  | sCJD-MM2:F16, 17,  sCJD-VV2:F16, F17 |
| 4 | CISY | O75390 | Citrate synthase, mitochondrial | Mc | Novel |  | sCJD-MM2:F12. 17,  sCJD-VV2:F16, 17 |
| 5 | STX1B | P61266 | Syntaxin-1B | Nu, C, Ck, Ce | Novel | Generalized epilepsy with febrile seizures plus 9 [38] | sCJD-MM2:F17,  sCJD-VV2:F16, 17 |
| 6 | PRIO | P04156 | Major prion protein (PrP) | Cm; Ga, Nu | Known [35] | Transmissible spongiform encephalopathies (Kuru, CJD, FFI and GSS), Alzheimer's disease [36] | sCJD-MM2:F17,  sCJD-VV2:F15 to F17 |
| 7 | CNRP1 | Q96F85 | CB1 cannabinoid receptor-interacting protein 1 |  | Novel |  | sCJD-MM2:F17,  sCJD-VV2:F17 |
| 8 | SCRN1 | Q12765 | Secernin-1 | C | Novel |  | sCJD-MM2:F17,  sCJD-VV2:F16, 17 |
| 9 | UBA1 | P22314 | Ubiquitin-like modifier-activating enzyme 1 | C, Mc, Nu | Novel |  | sCJD-MM2:F17,  sCJD-VV2:F16, 17 |
| 10 | AINX | Q16352 | Alpha-internexin |  | Novel |  | sCJD-MM2:F17,  sCJD-VV2:F16, 17 |
| 11 | PRDX5 | P30044 | Peroxiredoxin-5, mitochondrial | Mc, C, Px | Novel |  | sCJD-MM2:F17,  sCJD-VV2:F16 |

F12 to F17: HDF pool-12 to 17. Ce: centrosome, Sy: Synapse, Sr: sarcoplasmic reticulum, C: Cytoplasm, Ck: cytoskeleton, Nu: Nucleus, S: Secreted, Cm: Cell membrane, Sl: Sarcolemma, Ly: Lysosomes, Mc: Mitochondrion, Syo: Synaptosome, Cj: Cell junction, C V: cytoplasmic vesicles, Ga: Golgi apparatus, Pm: phagosome membrane, Px: peroxisome, Em: Endosome membrane, Cp: Cell projection, Gc: growth cone, Ms: Melanosome, Er: Endoplasmic reticulum and La: Lipid-anchor. The localization of proteins and accession number are assigned as in ExPASy protein database and Uniprot database respectively. Relevance with AD, prion and PrP ligand were established by Uniprot database search as well.

Certain HDP-interactors were unique to certain subtype-specific high-density fractions from each disease subtype cohort. The disease relevance and reported interaction to PrP are detailed in the **additional file 7**. We could also identify three interactors common to the controls and spAD HDFs including cathepsin D (CTSD), catenin beta-1 (CTNNB1) and protein piccolo (PCLO).

**Bibliography**

1. Yacoubian TA, Slone SR, Harrington AJ, Hamamichi S, Schieltz JM, Caldwell KA, et al. Differential neuroprotective effects of 14-3-3 proteins in models of Parkinson’s disease. Cell Death Dis. 2010;1:e2–e2.

2. Llorens F, Schmitz M, Knipper T, Schmidt C, Lange P, Fischer A, et al. Cerebrospinal Fluid Biomarkers of Alzheimer’s Disease Show Different but Partially Overlapping Profile Compared to Vascular Dementia. Front Aging Neurosci. 2017;9 SEP:289.

3. Schindler CK, Heverin M, Henshall DC. Isoform- and subcellular fraction-specific differences in hippocampal 14-3-3 levels following experimentally evoked seizures and in human temporal lobe epilepsy. J Neurochem. 2006;99:561–9.

4. Smith LM, Strittmatter SM. Binding Sites for Amyloid-β Oligomers and Synaptic Toxicity. Cold Spring Harb Perspect Med. 2017;7:a024075.

5. Querfurth HW, LaFerla FM. Alzheimer’s Disease. N Engl J Med. 2010;362:329–44.

6. Spiegel R, Pines O, Ta-Shma A, Burak E, Shaag A, Halvardson J, et al. Infantile Cerebellar-Retinal Degeneration Associated with a Mutation in Mitochondrial Aconitase, ACO2. Am J Hum Genet. 2012;90:518–23.

7. Zafar S, von Ahsen N, Oellerich M, Zerr I, Schulz-Schaeffer WJ, Armstrong VW, et al. Proteomics Approach to Identify the Interacting Partners of Cellular Prion Protein and Characterization of Rab7a Interaction in Neuronal Cells. J Proteome Res. 2011;10:3123–35.

8. Bogdanova N, Horst J, Chlystun M, Croucher PJP, Nebel A, Bohring A, et al. A common haplotype of the annexin A5 (ANXA5) gene promoter is associated with recurrent pregnancy loss. Hum Mol Genet. 2007;16:573–8.

9. Muramatsu T, Sakai N, Yanagihara I, Yamada M, Nishigaki T, Kokubu C, et al. Mutation analysis of the acid ceramidase gene in Japanese patients with Farber disease. J Inherit Metab Dis. 2002;25:585–92.

10. Huang Y, Tanimukai H, Liu F, Iqbal K, Grundke-Iqbal I, Gong C-X. Elevation of the level and activity of acid ceramidase in Alzheimer’s disease brain. Eur J Neurosci. 2004;20:3489–97.

11. Cha MY, Cho HJ, Kim C, Jung YO, Kang MJ, Murray ME, et al. Mitochondrial ATP synthase activity is impaired by suppressed O-GlcNAcylation in Alzheimer’s disease. Hum Mol Genet. 2015;24:6492–504.

12. Lin Q, Cao Y, Gao J. Serum calreticulin is a negative biomarker in patients with Alzheimer’s disease. Int J Mol Sci. 2014;15:21740–53.

13. Wang W, Chen R, Luo K, Wu D, Huang L, Huang T, et al. Calnexin inhibits thermal aggregation and neurotoxicity of prion protein. J Cell Biochem. 2010;111:343–9.

14. Kovacs GG, Sanchez-Juan P, Ströbel T, Schuur M, Poleggi A, Nocentini S, et al. Cathepsin D (C224T) Polymorphism in Sporadic and Genetic Creutzfeldt-Jakob Disease. Alzheimer Dis Assoc Disord. 2010;24:104–7.

15. Takenouchi T, Kosaki R, Niizuma T, Hata K, Kosaki K. Macrothrombocytopenia and developmental delay with a de novo CDC42 mutation: Yet another locus for thrombocytopenia and developmental delay. Am J Med Genet Part A. 2015;167:2822–5.

16. Weil D, D’Alessio M, Ramirez F, Eyre DR. Structural and functional characterization of a splicing mutation in the pro-alpha 2(I) collagen gene of an Ehlers-Danlos type VII patient. J Biol Chem. 1990;265:16007–11.

17. Jeanne M, Labelle-Dumais C, Jorgensen J, Kauffman WB, Mancini GM, Favor J, et al. COL4A2 Mutations Impair COL4A1 and COL4A2 Secretion and Cause Hemorrhagic Stroke. Am J Hum Genet. 2012;90:91–101.

18. Zafar S, Younas N, Sheikh N, Tahir W, Shafiq M, Schmitz M, et al. Cytoskeleton-Associated Risk Modifiers Involved in Early and Rapid Progression of Sporadic Creutzfeldt-Jakob Disease. Mol Neurobiol. 2017; June.

19. Abe S, Katagiri T, Saito-Hisaminato A, Usami S, Inoue Y, Tsunoda T, et al. Identification of CRYM as a candidate responsible for nonsyndromic deafness, through cDNA microarray analysis of human cochlear and vestibular tissues. Am J Hum Genet. 2003;72:73–82.

20. Ii K, Ito H, Kominami E, Hirano A. Abnormal distribution of cathepsin proteinases and endogenous inhibitors (cystatins) in the hippocampus of patients with Alzheimer’s disease, parkinsonism-dementia complex on Guam, and senile dementia and in the aged. Virchows Arch A Pathol Anat Histopathol. 1993;423:185–94.

21. Dianzani I, Howells DW, Ponzone A, Saleeba JA, Smooker PM, Cotton RG. Two new mutations in the dihydropteridine reductase gene in patients with tetrahydrobiopterin deficiency. J Med Genet. 1993;30:465–9.

22. Takito J, Yan L, Ma J, Hikita C, Vijayakumar S, Warburton D, et al. Hensin, the polarity reversal protein, is encoded by DMBT1, a gene frequently deleted in malignant gliomas. Am J Physiol. 1999;277 2 Pt 2:F277-89.

23. Valente L, Tiranti V, Marsano RM, Malfatti E, Fernandez-Vizarra E, Donnini C, et al. Infantile Encephalopathy and Defective Mitochondrial DNA Translation in Patients with Mutations of Mitochondrial Elongation Factors EFG1 and EFTu. Am J Hum Genet. 2007;80:44–58.

24. Horwitz M, Benson KF, Person RE, Aprikyan AG, Dale DC. Mutations in ELA2, encoding neutrophil elastase, define a 21-day biological clock in cyclic haematopoiesis. Nat Genet. 1999;23:433–6.

25. Comi GP, Fortunato F, Lucchiari S, Bordoni A, Prelle A, Jann S, et al. Beta-enolase deficiency, a new metabolic myopathy of distal glycolysis. Ann Neurol. 2001;50:202–7.

26. Gawinecka J, Cardone F, Asif AR, Pascalis A De, Wemheuer WM, Schulz-schaeffer WJ, et al. Sporadic Creutzfeldt – Jakob disease subtype-specific alterations of the brain proteome : Impact on Rab3a recycling. 2012;3610–20.

27. Schmitz-Abe K, Ciesielski SJ, Schmidt PJ, Campagna DR, Rahimov F, Schilke BA, et al. Congenital sideroblastic anemia due to mutations in the mitochondrial HSP70 homologue HSPA9. Blood. 2015;126:2734–8.

28. Au PYB, You J, Caluseriu O, Schwartzentruber J, Majewski J, Bernier FP, et al. GeneMatcher Aids in the Identification of a New Malformation Syndrome with Intellectual Disability, Unique Facial Dysmorphisms, and Skeletal and Connective Tissue Abnormalities Caused by De Novo Variants in HNRNPK. Hum Mutat. 2015;36:1009–14.

29. Bubber P, Haroutunian V, Fisch G, Blass JP, Gibson GE. Mitochondrial abnormalities in Alzheimer brain: Mechanistic implications. Ann Neurol. 2005;57:695–703.

30. Gawinecka J, Nowak M, Carimalo J, Cardone F, Asif AR, Wemheuer WM, et al. Subtype-specific synaptic proteome alterations in sporadic Creutzfeldt-Jakob disease. J Alzheimers Dis. 2013;37:51–61.

31. Hamdan FF, Srour M, Capo-Chichi J-M, Daoud H, Nassif C, Patry L, et al. De Novo Mutations in Moderate or Severe Intellectual Disability. PLoS Genet. 2014;10:e1004772.

32. Zafar S, Schmitz M, Younus N, Tahir W, Shafiq M, Llorens F, et al. Creutzfeldt-Jakob Disease Subtype-Specific Regional and Temporal Regulation of ADP Ribosylation Factor-1-Dependent Rho/MLC Pathway at Pre-Clinical Stage. J Mol Neurosci. 2015;56:329–48.

33. Zerr I, Schmitz M, Karch A, Villar-Piqué A, Kanata E, Golanska E, et al. Cerebrospinal fluid neurofilament light levels in neurodegenerative dementia: Evaluation of diagnostic accuracy in the differential diagnosis of prion diseases. Alzheimer’s Dement. 2018;14:751–63.

34. Weston PSJ, Poole T, Ryan NS, Nair A, Liang Y, Macpherson K, et al. Serum neurofilament light in familial Alzheimer disease. Neurology. 2017;89:2167–75.

35. Linden R, Martins VR, Prado MAM, Cammarota M, Izquierdo I, Brentani RR. Physiology of the Prion Protein. Physiol Rev. 2008;88:673–728.

36. Glatzel M, Stoeck K, Seeger H, Lührs T, Aguzzi A. Human prion diseases: Molecular and clinical aspects. Archives of Neurology. 2005;62:545–52.

37. Wu C-H, Fallini C, Ticozzi N, Keagle PJ, Sapp PC, Piotrowska K, et al. Mutations in the profilin 1 gene cause familial amyotrophic lateral sclerosis. Nature. 2012;488:499–503. doi:10.1038/nature11280.

38. Schubert J, Siekierska A, Langlois M, May P, Huneau C, Becker F, et al. Mutations in STX1B, encoding a presynaptic protein, cause fever-associated epilepsy syndromes. Nat Genet. 2014;46:1327–32. doi:10.1038/ng.3130.

39. Zafar S, Shafiq M, Younas N, Schmitz M, Ferrer I, Zerr I. Prion Protein Interactome: Identifying Novel Targets in Slowly and Rapidly Progressive Forms of Alzheimer’s Disease. J Alzheimer’s Dis. 2017;59:265–75. doi:10.3233/JAD-170237.

40. Leroy E, Boyer R, Auburger G, Leube B, Ulm G, Mezey E, et al. The ubiquitin pathway in Parkinson’s disease. Nature. 1998;395:451–2. doi:10.1038/26652.

41. Liu Y, Fallon L, Lashuel HA, Liu Z, Lansbury PT. The UCH-L1 gene encodes two opposing enzymatic activities that affect alpha-synuclein degradation and Parkinson’s disease susceptibility. Cell. 2002;111:209–18. http://www.ncbi.nlm.nih.gov/pubmed/12408865. Accessed 24 Jul 2018.

42. Setsuie R, Wada K. The functions of UCH-L1 and its relation to neurodegenerative diseases. Neurochem Int. 2007;51:105–11. doi:10.1016/j.neuint.2007.05.007.

43. Vesa J, Hellsten E, Verkruyse LA, Camp LA, Rapola J, Santavuori P, et al. Mutations in the palmitoyl protein thioesterase gene causing infantile neuronal ceroid lipofuscinosis. Nature. 1995;376:584–7. doi:10.1038/376584a0.

1. Yacoubian TA, Slone SR, Harrington AJ, Hamamichi S, Schieltz JM, Caldwell KA, et al. Differential neuroprotective effects of 14-3-3 proteins in models of Parkinson’s disease. Cell Death Dis. 2010;1:e2–e2. doi:10.1038/cddis.2009.4.

2. Llorens F, Schmitz M, Knipper T, Schmidt C, Lange P, Fischer A, et al. Cerebrospinal Fluid Biomarkers of Alzheimer’s Disease Show Different but Partially Overlapping Profile Compared to Vascular Dementia. Front Aging Neurosci. 2017;9 SEP:289. doi:10.3389/fnagi.2017.00289.

3. Schindler CK, Heverin M, Henshall DC. Isoform- and subcellular fraction-specific differences in hippocampal 14-3-3 levels following experimentally evoked seizures and in human temporal lobe epilepsy. J Neurochem. 2006;99:561–9. doi:10.1111/j.1471-4159.2006.04153.x.

4. Smith LM, Strittmatter SM. Binding Sites for Amyloid-β Oligomers and Synaptic Toxicity. Cold Spring Harb Perspect Med. 2017;7:a024075. doi:10.1101/cshperspect.a024075.

5. Querfurth HW, LaFerla FM. Alzheimer’s Disease. N Engl J Med. 2010;362:329–44. doi:10.1056/NEJMra0909142.

6. Spiegel R, Pines O, Ta-Shma A, Burak E, Shaag A, Halvardson J, et al. Infantile Cerebellar-Retinal Degeneration Associated with a Mutation in Mitochondrial Aconitase, ACO2. Am J Hum Genet. 2012;90:518–23. doi:10.1016/j.ajhg.2012.01.009.

7. Zafar S, von Ahsen N, Oellerich M, Zerr I, Schulz-Schaeffer WJ, Armstrong VW, et al. Proteomics Approach to Identify the Interacting Partners of Cellular Prion Protein and Characterization of Rab7a Interaction in Neuronal Cells. J Proteome Res. 2011;10:3123–35. doi:10.1021/pr2001989.

8. Bogdanova N, Horst J, Chlystun M, Croucher PJP, Nebel A, Bohring A, et al. A common haplotype of the annexin A5 (ANXA5) gene promoter is associated with recurrent pregnancy loss. Hum Mol Genet. 2007;16:573–8. doi:10.1093/hmg/ddm017.

9. Muramatsu T, Sakai N, Yanagihara I, Yamada M, Nishigaki T, Kokubu C, et al. Mutation analysis of the acid ceramidase gene in Japanese patients with Farber disease. J Inherit Metab Dis. 2002;25:585–92. http://www.ncbi.nlm.nih.gov/pubmed/12638942. Accessed 23 Jul 2018.

10. Huang Y, Tanimukai H, Liu F, Iqbal K, Grundke-Iqbal I, Gong C-X. Elevation of the level and activity of acid ceramidase in Alzheimer’s disease brain. Eur J Neurosci. 2004;20:3489–97. doi:10.1111/j.1460-9568.2004.03852.x.

11. Cha MY, Cho HJ, Kim C, Jung YO, Kang MJ, Murray ME, et al. Mitochondrial ATP synthase activity is impaired by suppressed O-GlcNAcylation in Alzheimer’s disease. Hum Mol Genet. 2015;24:6492–504. doi:10.1093/hmg/ddv358.

12. Lin Q, Cao Y, Gao J. Serum calreticulin is a negative biomarker in patients with Alzheimer’s disease. Int J Mol Sci. 2014;15:21740–53. doi:10.3390/ijms151221740.

13. Wang W, Chen R, Luo K, Wu D, Huang L, Huang T, et al. Calnexin inhibits thermal aggregation and neurotoxicity of prion protein. J Cell Biochem. 2010;111:343–9. doi:10.1002/jcb.22698.

14. Kovacs GG, Sanchez-Juan P, Ströbel T, Schuur M, Poleggi A, Nocentini S, et al. Cathepsin D (C224T) Polymorphism in Sporadic and Genetic Creutzfeldt-Jakob Disease. Alzheimer Dis Assoc Disord. 2010;24:104–7. doi:10.1097/WAD.0b013e3181ad378c.

15. Takenouchi T, Kosaki R, Niizuma T, Hata K, Kosaki K. Macrothrombocytopenia and developmental delay with a de novo CDC42 mutation: Yet another locus for thrombocytopenia and developmental delay. Am J Med Genet Part A. 2015;167:2822–5. doi:10.1002/ajmg.a.37275.

16. Weil D, D’Alessio M, Ramirez F, Eyre DR. Structural and functional characterization of a splicing mutation in the pro-alpha 2(I) collagen gene of an Ehlers-Danlos type VII patient. J Biol Chem. 1990;265:16007–11. http://www.ncbi.nlm.nih.gov/pubmed/2394758. Accessed 23 Jul 2018.

17. Jeanne M, Labelle-Dumais C, Jorgensen J, Kauffman WB, Mancini GM, Favor J, et al. COL4A2 Mutations Impair COL4A1 and COL4A2 Secretion and Cause Hemorrhagic Stroke. Am J Hum Genet. 2012;90:91–101. doi:10.1016/j.ajhg.2011.11.022.

18. Zafar S, Younas N, Sheikh N, Tahir W, Shafiq M, Schmitz M, et al. Cytoskeleton-Associated Risk Modifiers Involved in Early and Rapid Progression of Sporadic Creutzfeldt-Jakob Disease. Mol Neurobiol. 2017; June. doi:10.1007/s12035-017-0589-0.

19. Abe S, Katagiri T, Saito-Hisaminato A, Usami S, Inoue Y, Tsunoda T, et al. Identification of CRYM as a candidate responsible for nonsyndromic deafness, through cDNA microarray analysis of human cochlear and vestibular tissues. Am J Hum Genet. 2003;72:73–82. http://www.ncbi.nlm.nih.gov/pubmed/12471561. Accessed 23 Jul 2018.

20. Ii K, Ito H, Kominami E, Hirano A. Abnormal distribution of cathepsin proteinases and endogenous inhibitors (cystatins) in the hippocampus of patients with Alzheimer’s disease, parkinsonism-dementia complex on Guam, and senile dementia and in the aged. Virchows Arch A Pathol Anat Histopathol. 1993;423:185–94. http://www.ncbi.nlm.nih.gov/pubmed/8236812. Accessed 23 Jul 2018.

21. Dianzani I, Howells DW, Ponzone A, Saleeba JA, Smooker PM, Cotton RG. Two new mutations in the dihydropteridine reductase gene in patients with tetrahydrobiopterin deficiency. J Med Genet. 1993;30:465–9. http://www.ncbi.nlm.nih.gov/pubmed/8326489. Accessed 23 Jul 2018.

22. Takito J, Yan L, Ma J, Hikita C, Vijayakumar S, Warburton D, et al. Hensin, the polarity reversal protein, is encoded by DMBT1, a gene frequently deleted in malignant gliomas. Am J Physiol. 1999;277 2 Pt 2:F277-89. http://www.ncbi.nlm.nih.gov/pubmed/10444583. Accessed 23 Jul 2018.

23. Valente L, Tiranti V, Marsano RM, Malfatti E, Fernandez-Vizarra E, Donnini C, et al. Infantile Encephalopathy and Defective Mitochondrial DNA Translation in Patients with Mutations of Mitochondrial Elongation Factors EFG1 and EFTu. Am J Hum Genet. 2007;80:44–58. doi:10.1086/510559.

24. Horwitz M, Benson KF, Person RE, Aprikyan AG, Dale DC. Mutations in ELA2, encoding neutrophil elastase, define a 21-day biological clock in cyclic haematopoiesis. Nat Genet. 1999;23:433–6. doi:10.1038/70544.

25. Comi GP, Fortunato F, Lucchiari S, Bordoni A, Prelle A, Jann S, et al. Beta-enolase deficiency, a new metabolic myopathy of distal glycolysis. Ann Neurol. 2001;50:202–7. http://www.ncbi.nlm.nih.gov/pubmed/11506403. Accessed 24 Jul 2018.

26. Gawinecka J, Cardone F, Asif AR, Pascalis A De, Wemheuer WM, Schulz-schaeffer WJ, et al. Sporadic Creutzfeldt – Jakob disease subtype-specific alterations of the brain proteome : Impact on Rab3a recycling. 2012;:3610–20.

27. Schmitz-Abe K, Ciesielski SJ, Schmidt PJ, Campagna DR, Rahimov F, Schilke BA, et al. Congenital sideroblastic anemia due to mutations in the mitochondrial HSP70 homologue HSPA9. Blood. 2015;126:2734–8. doi:10.1182/blood-2015-09-659854.

28. Au PYB, You J, Caluseriu O, Schwartzentruber J, Majewski J, Bernier FP, et al. GeneMatcher Aids in the Identification of a New Malformation Syndrome with Intellectual Disability, Unique Facial Dysmorphisms, and Skeletal and Connective Tissue Abnormalities Caused by De Novo Variants in HNRNPK. Hum Mutat. 2015;36:1009–14. doi:10.1002/humu.22837.

29. Bubber P, Haroutunian V, Fisch G, Blass JP, Gibson GE. Mitochondrial abnormalities in Alzheimer brain: Mechanistic implications. Ann Neurol. 2005;57:695–703. doi:10.1002/ana.20474.

30. Gawinecka J, Nowak M, Carimalo J, Cardone F, Asif AR, Wemheuer WM, et al. Subtype-specific synaptic proteome alterations in sporadic Creutzfeldt-Jakob disease. J Alzheimers Dis. 2013;37:51–61. doi:10.3233/JAD-130455.

31. Hamdan FF, Srour M, Capo-Chichi J-M, Daoud H, Nassif C, Patry L, et al. De Novo Mutations in Moderate or Severe Intellectual Disability. PLoS Genet. 2014;10:e1004772. doi:10.1371/journal.pgen.1004772.

32. Zafar S, Schmitz M, Younus N, Tahir W, Shafiq M, Llorens F, et al. Creutzfeldt-Jakob Disease Subtype-Specific Regional and Temporal Regulation of ADP Ribosylation Factor-1-Dependent Rho/MLC Pathway at Pre-Clinical Stage. J Mol Neurosci. 2015;56:329–48. doi:10.1007/s12031-015-0544-3.

33. Zerr I, Schmitz M, Karch A, Villar-Piqué A, Kanata E, Golanska E, et al. Cerebrospinal fluid neurofilament light levels in neurodegenerative dementia: Evaluation of diagnostic accuracy in the differential diagnosis of prion diseases. Alzheimer’s Dement. 2018;14:751–63. doi:10.1016/j.jalz.2017.12.008.

34. Weston PSJ, Poole T, Ryan NS, Nair A, Liang Y, Macpherson K, et al. Serum neurofilament light in familial Alzheimer disease. Neurology. 2017;89:2167–75. doi:10.1212/WNL.0000000000004667.

35. Linden R, Martins VR, Prado MAM, Cammarota M, Izquierdo I, Brentani RR. Physiology of the Prion Protein. Physiol Rev. 2008;88:673–728. doi:10.1152/physrev.00007.2007.

36. Glatzel M, Stoeck K, Seeger H, Lührs T, Aguzzi A. Human prion diseases: Molecular and clinical aspects. Archives of Neurology. 2005;62:545–52.

37. Wu C-H, Fallini C, Ticozzi N, Keagle PJ, Sapp PC, Piotrowska K, et al. Mutations in the profilin 1 gene cause familial amyotrophic lateral sclerosis. Nature. 2012;488:499–503. doi:10.1038/nature11280.

38. Schubert J, Siekierska A, Langlois M, May P, Huneau C, Becker F, et al. Mutations in STX1B, encoding a presynaptic protein, cause fever-associated epilepsy syndromes. Nat Genet. 2014;46:1327–32. doi:10.1038/ng.3130.

39. Zafar S, Shafiq M, Younas N, Schmitz M, Ferrer I, Zerr I. Prion Protein Interactome: Identifying Novel Targets in Slowly and Rapidly Progressive Forms of Alzheimer’s Disease. J Alzheimer’s Dis. 2017;59:265–75. doi:10.3233/JAD-170237.

40. Leroy E, Boyer R, Auburger G, Leube B, Ulm G, Mezey E, et al. The ubiquitin pathway in Parkinson’s disease. Nature. 1998;395:451–2. doi:10.1038/26652.

41. Liu Y, Fallon L, Lashuel HA, Liu Z, Lansbury PT. The UCH-L1 gene encodes two opposing enzymatic activities that affect alpha-synuclein degradation and Parkinson’s disease susceptibility. Cell. 2002;111:209–18. http://www.ncbi.nlm.nih.gov/pubmed/12408865. Accessed 24 Jul 2018.

42. Setsuie R, Wada K. The functions of UCH-L1 and its relation to neurodegenerative diseases. Neurochem Int. 2007;51:105–11. doi:10.1016/j.neuint.2007.05.007.

43. Vesa J, Hellsten E, Verkruyse LA, Camp LA, Rapola J, Santavuori P, et al. Mutations in the palmitoyl protein thioesterase gene causing infantile neuronal ceroid lipofuscinosis. Nature. 1995;376:584–7. doi:10.1038/376584a0.
